# Validation of the French Version of the Positivity Scale (P Scale)

**DOI:** 10.3389/fpsyg.2021.724253

**Published:** 2022-02-02

**Authors:** Alexis Vancappel, Robert Courtois, Marta Siragusa, Coraline Hingray, Christian Réveillère, Gianvittorio Caprara, Catherine Belzung, Wissam El-Hage

**Affiliations:** ^1^Pôle de Psychiatrie-Addictologie, CHRU de Tours, Tours, France; ^2^Département de Psychologie, EE 1901 Qualipsy, Qualité de vie et Santé Psychologique, Université de Tours, Tours, France; ^3^UMR 1253, iBrain, Inserm, Université de Tours, Tours, France; ^4^Pôle Hospitalo-Universitaire de Psychiatrie d’Adultes et d’Addictologie du Grand Nancy, Centre Psychothérapique de Nancy (CPN), Laxou, France; ^5^Department of Psychology, Sapienza University of Rome, Rome, Italy

**Keywords:** positivity, well-being, self-esteem, cognitive, psychometric

## Abstract

**Background:**

The purpose of this study is to assess the psychometric properties of the French version of the Positivity scale (P scale), a self-report measure of positivity, which is the tendency to view and address life and experience with a positive outlook. Positivity is seen as a latent factor underlying multiple cognitive concepts such as self-esteem, life satisfaction, and optimism.

**Methods:**

We recruited 666 volunteers (540 women and 126 men). They completed the P scale online, as well as self-report measures of psychological well-being, self-esteem, satisfaction with life, general health, and personality dimensions. The study sample was randomly split into two sub-groups, one used for exploratory factor analysis and the other for confirmatory factor analysis.

**Results:**

We found conflictual results related to the uni-dimensionality of the French version of the P scale. We found good internal consistency and high concurrent validity.

**Conclusion:**

The French version of the P scale demonstrated good psychometric qualities and is a reliable tool that can now be used by French researchers and clinicians to assess positivity.

## Background

### Positivity and P Scale

Positivity is defined as a propensity to evaluate aspects of life in general as good (Diener et al., 2000). To evaluate this concept, authors developed the Positivity Scale (P scale) (Caprara et al., 2012), an 8-item questionnaire demonstrating excellent psychometric properties, notably concurrent validity. Its internal consistency is good (α Cronbach = 0.75), with good test–retest stability (0.76). The P scale has also shown excellent psychometric properties in Italian, English, Spanish, and Japanese, suggesting that the positivity construct is common across different cultural contexts (Caprara et al., 2012). The P scale can be used with people of all ages. For example, it has been validated in late childhood/early adolescence, in a study that also confirmed its one-factor structure (Zuffianò et al., 2019). Another study found that positivity plays an important role in the elderly (62–80 years old) (Caprara et al., 2017b).

### Positivity as a Common Factor for Self-Esteem, Optimism and Life Satisfaction

Positivity seems to be the common factor of three psychological concepts: self-esteem, optimism and life satisfaction. Self-esteem, conceptualized as a global evaluation of the self (Sowislo and Orth, 2013), shown to be positively linked to well-being (Lin, 2015) and negatively linked to dysphoria (Remue et al., 2014) and depression (Franck et al., 2007). Optimism is defined as “the belief that good, as opposed to bad, things will generally occur in one’s life” (Scheier and Carver, 1993). Studies have also shown a negative relationship between optimism and dysphoria (Scheier et al., 1994) and a positive relationship with coping skills and with mental and physical well-being (Conversano et al., 2010). Life satisfaction refers to the overall evaluation of one’s quality of life and corresponds to the cognitive component of well-being (Diener et al., 1985). Satisfaction with life has been shown to be negatively related to dysphoria (Guney et al., 2010). A large cross-sectional study has set out that there is a common factor underlying these three concepts (self-esteem, life satisfaction, and optimism), arguing in favor of the existence of positivity (Alessandri et al., 2012a).

### Positivity Across the Different Fields of Psychology

As suggested by a recent meta-analysis, the development of the P scale has revealed positive relationships between positivity and other psychological concepts in different fields (Caprara et al., 2019). First, studies in organizational contexts found a positive association between positivity and job performance (Alessandri et al., 2012b; Livi et al., 2015), work engagement (Alessandri et al., 2015), work/life balance, and job satisfaction (Orkibi and Brandt, 2015). Positivity has also been found to be linked to social behavior, with positive correlations with prosocial behavior and negative correlations with internalizing and externalizing problems (Zuffianò et al., 2019). Another study found a positive association between positivity and family life goals (Laguna et al., 2017).

Other studies have established links between positivity and general health or adjustment to health problems. For example, it has been shown to be associated with reduced functional impairment in cancer patients (Caprara et al., 2016) and with better functioning of the immune system (Caprara et al., 2017b). However, the authors suggested that positivity may not affect health problems directly, but rather attenuate the way they are perceived and decrease anxiety about the future (Caprara et al., 2017b). Finally, it has been suggested that positivity may play a role in mental health. For example, Beck proposed that negative thoughts are involved in the onset and maintenance of depression (Beck, 1979, 2008), while, conversely, Caprara et al. (2010) suggested that positivity provides protection from depression. Empirical data have shown that positivity is associated with general positive affects (Alessandri et al., 2014; Caprara et al., 2017a). To strengthen this idea, positivity has been found as related to the traits of the big five inventory (BFI) (Agreeableness, Consciousness, Neuroticism, and Openness) (Caprara et al., 2012). Indeed, neuroticism is defined as a trait disposition to experience negative affects, including anger, anxiety, irritability, emotional instability, and depression (Leary and Hoyle, 2009). It can be understood as an opposed dimension to positivity. More generally, the positive traits of the BFI have been identified as positively related to positive emotions (Shiota et al., 2006).

### Objective and Hypothesis

Thus, it seems important to have a self-assessment tool for positivity with good psychometric properties, such as the P scale. This would allow the use of this scale for further studies and clinical practice. The purpose of the present study was to assess the psychometric properties of the French version of the P scale, and to examine its internal consistency, convergent validity, and factor structure. We hypothesized a one-factor structure of the P scale, as proposed by previous research. We hypothesized that Positivity would be positively related to quality of life, self-esteem, agreeability, and openness, and negatively related to general health problems and neuroticism. We did not evaluate the association between the P scale and optimism as it is not necessary to assess the concurrent validity of the P scale and as optimism has been less studied than the other concepts used for such assessment.

## Materials and Methods

### Participants

Participation required reading an information note online, ticking a box to consent to participate, and choosing to either continue with the study or decline to proceed. The study and consent procedures were approved by the ethics committee of the University (Comité d’Ethique de la Recherche Tours-Poitiers, 2019-02-03). Participants did not receive any reward for participating in the study. The software did not gather incomplete responses.

We recruited 666 participants (540 women, 126 men) through social networks and small posters at the university. Their mean age was 27.44 years (10.96); 54.8% of the participants were single and 45.20% were in a relationship. Most of the participants (66.67%) were students, 27.33% were employed and 3.3% were unemployed. All statistical analyses were performed on the whole sample, apart from factor analyses, for which the sample was split into two random sub-groups, with similar characteristics. There were 333 participants in each of these groups, 272 women (mean age 27.45 ± 11.50) in the first, and 268 in the second (mean age 27.43 ± 1.41). Descriptive statistics of the whole sample are displayed in Table 1. Sociodemographic information were gathered with multiple choices question and numerical scales.

**TABLE 1:** Descriptive data of overall scale scores.

	Mean	Minimum	Maximum	SD
**Quality of life**				
Quality of life scale	24.71	5.00	35.00	6.39
**Positivity**				
Positivity scale	28.00	9.00	40.00	6.12
**Self-esteem**				
Rosenberg self-esteem scale	29.71	12.00	40.00	5.91
**General health**				
Somatization-GHQ	6.50	0.00	20.00	4.20
Anxiety Insomnia-GHQ	7.91	0.00	21.00	5.08
Social dysfunction-GHQ	8.51	1.00	21.00	3.33
Depression-GHQ	3.48	0.00	21.00	4.53
**Personality dimensions**				
Extraversion-BFI	3.13	1.13	5.00	0.89
Agreeableness-BFI	3.94	1.90	5.00	0.56
Consciousness-BFI	3.64	1.56	5.00	0.73
Neuroticism-BFI	3.18	1.00	5.00	0.96
Openness-BFI	3.65	1.50	5.00	0.68

*GHQ, general health questionnaire; BFI, big five inventory; SD, Standard deviation.*

### Measures

After providing their informed consent, participants completed a series of online questionnaires.

#### The Positivity Scale

The original Italian questionnaire (Caprara et al., 2012) was translated into French by a member of the research team (CB) whose mother tongue is French but who is also fluent in Italian. The French version was then back-translated by an independent translator blinded to the original version. The back-translated version was submitted to the developer of the original Italian P scale (Caprara). Minor changes were agreed and the pre-final form was modified to include these changes. This version became the final French version of the P scale for validation testing (Supplementary Material 1). The P scale has eight items evaluating positivity, in other words, the tendency to see life and experiences positively, for example, “I have great faith in the future.” Respondents rate their level of agreement with each item on a five-point scale from 1 (strongly disagree) to 5 (strongly agree), higher scores reflecting greater positivity. The original version has been shown to have good internal consistency (α = 0.75) (Caprara et al., 2012).

#### Rosenberg Self-Esteem Scale

This is a self-report questionnaire (Rosenberg, 1965) that evaluates self-worth through 10 items, measuring both positive and negative feelings about oneself; for example, “On the whole, I am satisfied with myself.” The participants rate each item on a Likert scale ranging from 1 (strongly agree) to 4 (strongly disagree); some items are reverse scored. Higher scores indicate higher self-esteem. The French version has shown good psychometric properties, and notably good internal consistency (between 0.70 and 0.90) (Vallieres and Vallerand, 1990).

#### General Health Questionnaire

The general health questionnaire (GHQ) (Goldberg and Blackwell, 1970) is a 28-item questionnaire, frequently used as an indicator of mental health. It covers four dimensions: depression, anxiety-insomnia, social dysfunction, somatization. Items are rated on a Likert scale from 1 (not at all) to 4 (much more than usual). The total score ranges from 28 to 112; high scores indicate poor psychological well-being. The French version has shown good psychometric properties (α = 0.91) (Pariente et al., 1992).

#### Satisfaction With Life Scale

This five-item self-report questionnaire (Diener et al., 1985) evaluates overall life satisfaction (e.g., “In most ways my life is close to my ideal”). Each item is rated on a seven-point Likert scale, from 1 (strongly disagree) to 7 (strongly agree). The satisfaction with life scale (SWLS) score is the sum of the item scores, ranging from 5 (lowest satisfaction) to 35 (highest satisfaction). We used the French version of the SWLS, which has excellent psychometric properties and good internal consistency (α = 0.80) (Blais et al., 1989).

#### Big Five Inventory

The BFI is a 45-item questionnaire (John et al., 1991) that evaluates five dimensions of personality, namely Extraversion, Agreeableness, Conscientiousness, Neuroticism, and Openness. Items are rated on a five-point Likert scale ranging from 1 (strongly disagree) to 5 (strongly agree). The French version has demonstrated good psychometric properties, notably good internal consistency for all the dimensions (Cronbach’s α between 0.74 and 0.82) (Plaisant et al., 2010).

### Statistical Analyses

As recommended by authors (Orcan, 2018), we randomly split the general population in two sub-groups to perform exploratory and confirmatory factor analysis (CFA). We performed an exploratory factor analysis (EFA) on the first sub-group and a CFA on the second to confirm the one-factor structure of positivity found by Caprara et al. (2012). Following previous guidelines for EFA (Cattell, 1966), we excluded factors behind the eigenvalue collapse. We considered a factor loading greater than 0.30 as acceptable (Costello and Osborne, 2005). We also considered communalities, with indexes greater than 0.20, considered as acceptable (Child, 2006). We used Horn’s parallel analysis to determine the relevant number of factors (Horn, 1965). Concerning CFA, as proposed by authors (Tanaka, 1987), χ^2^ can be significant, but cannot lead to the rejection of the model. Therefore, to assess the fit of the model, we used comparative fit index (CFI) and tucker-lewis index (TLI), with values above 0.90 indicating an acceptable fit of the model and values above 0.95 indicating an good fit of the model (Browne and Cudeck, 1992; Hu and Bentler, 1999; Gana and Broc, 2018). We also calculated the Root Mean Square Error of Approximation (RMSEA), values below 0.06 indicating a good fit of the model (Browne and Cudeck, 1992; Hu and Bentler, 1999; Gana and Broc, 2018), as well as χ^2^/df, values above 2 (Byrne, 2011, 2016). We first assessed a one-factor structure model without any correlations between errors. Then, we added correlations between errors based on modification indices (items 1 and 4, items 4 and 6, items 6 and 1). Indeed, researchers claim “correlated errors are possible among items using similar wording or appearing near to each other on the questionnaire.” (Bollen and Lennox, 1991), which is true for items 1, 4, and 6. We evaluated invariance by comparing the model fit indexes with and without equality constraints. We considered χ^2^ differences’ significance and ΔCFI above 0.001 as criterion to reject invariance (Cheung and Rensvold, 2002). Concerning the age criteria, the groups computed for the analysis were created based on the median score.

We evaluated the internal consistency using Cronbach’s alpha, with a score greater than 0.80, indicating a good internal consistency (Cronbach, 1965). In accordance with the most recent guidelines, we used McDonald’s Omega to complete this evaluation (Hayes and Coutts, 2020). Indeed, Omega avoids false conclusion due to inappropriate assumptions usually used for Cronbach’s value such as equal sensitivity across items. The omega was calculated using Hayes and Coutts (2020) SPSS plugging. We also evaluated inter-item correlations.

Finally, we performed correlational analyses to evaluate the concurrent validity of the P scale. We assessed the correlation between positivity and other concepts. To avoid the overestimation of the relationship between positivity and other concepts because of the redundant items, we performed another correlation analysis with item 7 removed from the global score of rosenberg self-esteem scale (RSES). This item was redundant with Positivity item 5. Statistical analyses were performed using SPSS 23 and AMOS 23 software.

## Results

### Factor Structure

We assessed inter-item correlations and found that all items were significantly correlated to each other, with a *p*-value under 0.05. The results are displayed in Table 2.

**TABLE 2:** Inter-item correlations.

	Item 1	Item 2	Item 3	Item 4	Item 5	Item 6	Item 7	Item 8
**Item 1**	1	0.46	0.22	0.71	0.44	0.51	0.34	0.42
**Item 2**		1	0.41	0.49	0.56	0.29	0.44	0.37
**Item 3**			1	0.28	0.33	0.15	0.28	0.17
**Item 4**				1	0.48	0.47	0.37	0.37
**Item 5**					1	0.30	0.60	0.56
**Item 6**						1	0.23	0.31
**Item 7**							1	0.48
**Item 8**								1

*All correlations are significant, p-value < 0.001.*

We conducted an EFA on the first sub-group. The Kaiser-Meyer-Olkin test (KMO) yielded a value of 0.84 indicating good sampling adequacy of the model. Bartlett’s test was also significant (χ^2^ = 966.915; df = 28; *p* < 0.001). The factor loadings and communalities are presented in Table 3. We carried out a principal axis factor analysis using Varimax rotation on the data obtained from the responses to the eight items of the P scale. The results confirmed the one-factor structure with eigenvalues ranging from 3.78 for the first factor (accounting for 47.28% of the global variance) to 1.12 for the second factor (accounting for 13.96% of the global variance). The score of the first factor was above the random score proposed by the parallel analysis (1.24). However, the score of the second factor was under the random score proposed by the same analysis (1.15). This argue in favor of the one-factor structure.

**TABLE 3:** Results of exploratory factor analysis performed on the first sub-group.

	Items	Factor loadings	Communality
4.	I look forward to the future with hope and enthusiasm	0.76	0.57
1.	I have great faith in the future	0.75	0.56
5.	On the whole, I am satisfied with myself	0.71	0.50
2.	I am satisfied with my life	0.68	0.46
7.	I feel I have many things to be proud of	0.58	0.33
8.	I generally feel confident in myself	0.56	0.31
3.	Others are generally here for me when I need them	0.50	0.25
6.	At times, the future seems unclear to me (reverse scored)	0.47	0.22
Eigenvalue		3.78	
Explained variance		0.47	

For CFA, we assessed a one-factor structure model without any correlations between items error. χ^2^ was significant (χ^2^ = 187.62, df = 20, *p* < 0.001), but this cannot lead to rejection of the model due to the important size of the sample. The other indexes did not show a good fit of the model (CFI = 0.84; TLI = 0.77; RMSEA = 0.159, χ^2^/df = 187.620/20 = 9.38). Then, we evaluated the one-factor structure model, adding correlations between the three more related errors. The correlations are between the errors of items 1 and 4, 1 and 6, and 6 and 4. We found a good model fit (CFI = 0.976; TLI = 0.962; RMSEA = 0.037, χ^2^/df = 54.36/52 = 1.45). The standardized estimates of the CFA are presented in Table 4. Finally, we assessed invariance. We found invariance for age (χ^2^ diff = 187.62, df = 7; *p* > 0.3; ΔCFI = 0.001). However we did not find invariance for sex (χ^2^ diff = 20.76, df = 7; *p* < 0.01; ΔCFI = 0.013) and for socio-professional level (χ^2^ diff = 30.54, df = 13; *p* < 0.01; ΔCFI = 0.018).

**TABLE 4:** Results of confirmatory factor analysis in the second sub-group.

	Items	Standardized estimates
5.	On the whole, I am satisfied with myself	0.876
7.	I feel I have many things to be proud of	0.711
2.	I am satisfied with my life	0.708
8.	I generally feel confident in myself	0.703
4.	I look forward to the future with hope and enthusiasm	0.593
1.	I have great faith in the future	0.515
6.	At times, the future seems unclear to me (reverse scored)	0.418
3.	Others are generally here for me when I need them	0.326

### Internal Consistency

We evaluated the internal consistency in the global sample and found that the reliability of the French P scale is satisfactory with high internal consistency (Cronbach α = 0.84 and McDonald’s ω = 0.84).

### Concurrent Validity

We observed that positivity scores were significantly and positively correlated with self-esteem and quality of life. They were also positively correlated with Extraversion, Agreeableness, Conscientiousness, and Openness, and negatively correlated with Neuroticism and the general health sub-dimensions of somatization, anxiety-insomnia, social dysfunction and depression. All results are presented in Table 5. To examine redundant items between the P scale and Rosenberg’s self-esteem scale, we removed identical items and performed correlation analysis between the P scale and the revised RSES (see Table 5).

**TABLE 5:** Correlations of positivity (P scale score) with other constructs.

	Positivity
**Quality of life**	
Satisfaction with life scale	0.68**
**Self-esteem**	
Rosenberg self-esteem scale	0.78**
Revised-Rosenberg self-esteem scale	0.75**
**General health**	
Somatization-GHQ	−0.32**
Anxiety insomnia-GHQ	−0.42**
Social dysfunction-GHQ	−0.41**
Depression-GHQ	−0.60**
**Personality dimensions**	
Extraversion-BFI	0.40*
Agreeableness-BFI	0.24**
Conscientiousness-BFI	0.44**
Neuroticism-BFI	−0.54**
Openness-BFI	0.21**

** p-value < 0.001. GHQ, general health questionnaire; BFI, big five inventory.

## Discussion

This study examined the psychometric properties of a new French version of the P scale; results show that it has good internal consistency. Factor analysis also partially confirmed the one-factor structure of the scale, as found by Caprara et al. (2012) in the original version. Indeed, parallel analysis tend to confirm the one-factor structure, however, the model fit indexes found in the CFA are weak. These indexes are lower than those identified in Caprara et al. (2012). We also did not succeed to validate invariance for sex and socio-professional level. One first explanation may be the important correlation between items 1 and 4 that creates a correlation between errors of those two items. Another explanation may be that self-esteem, optimism and quality of life are influenced by other mechanisms and are not related to the only concept of positivity within the French population. Concurrent validity was established. Overall, the results indicate that the French P scale is a psychometrically sound instrument that can be used by French clinicians and researchers.

The strong correlations obtained between positivity and self-esteem, and between positivity and quality of life, are close to those obtained in the initial version by Caprara et al. (2012). These strong correlations were expected given that positivity is assumed to be an underlying factor common to self-worth and quality of life (Caprara et al., 2010). The results obtained in this study also confirm the negative relationship between positivity and emotional distress, including anxiety, depression, and neuroticism.

The development of the P scale is of great interest in psychotherapy, especially for cognitive behavior therapy (CBT), in which clinicians often use cognitive restructuring to change the pattern of thinking underlying people’s difficulties. Different models have been developed for specific disorders, such as depression (King, 2002), social phobia (Clark and Wells, 1995), panic disorder (Clark, 1986), eating disorders (Fairburn et al., 2003), post-traumatic stress disorder (Ehlers and Clark, 2000), and generalized anxiety disorder (Wells, 1999). According to Caprara et al. (2019), positivity is a factor underlying many outcomes, suggesting the relevance of helping patients modify their general vision of the world instead of focusing on specific thoughts. Taking this line, positivity could be considered as a transdiagnostic process; developing positive thoughts through psychotherapy could reduce the symptoms of multiple disorders and increase positive outcomes such as social interactions, work success, and physical health.

The development of the P scale is also of great interest in health and organizational psychology. Previous studies have highlighted the positive relationship between positivity and job performance (Alessandri et al., 2012b; Livi et al., 2015) and work/life balance (Orkibi and Brandt, 2015). It has also been shown that positivity can reduce functional impairment in serious illnesses such as cancer (Caprara et al., 2016) and improve the functioning of the immune system (Caprara et al., 2017b), highlighting its protective role. Further research is now needed to determine the malleability of positivity and how it can some be used in psychiatry, organizational psychology, and health psychology.

This study has some limitations. We examined the correlational relationship between positivity and emotional distress, and further experimental studies are needed to determine its causal role. There was also an important imbalance between men and women in our sample, limiting the conclusions that can be drawn. Indeed, 81% of the participants are women. Moreover, participants were mostly students. Further studies assessing the psychometric properties of the P scale should be performed with general population groups. Moreover, we did not examine the psychometric properties of the P scale in a psychiatric population, and it would be interesting to examine the role of positivity in emotional disorders. Further studies including test–retest reliability are also required. Finally, as we used self-report measures, the results can be influenced by social desirability.

## Conclusion

This is the first study presenting a French version of the P scale, which is a self-report questionnaire showing good psychometric qualities. It is a reliable instrument that can be used to identify an individual’s tendency to have a positive outlook on life and experience. The simplicity and rapidity of completing the P scale makes it suitable for use in clinical practice and in many research domains.

## Data Availability Statement

The raw data supporting the conclusions of this article will be made available by the authors, without undue reservation.

## Ethics Statement

The studies involving human participants were reviewed and approved by Comité d’Ethique de la Recherche Tours-Poitiers. The patients/participants provided their written informed consent to participate in this study.

## Author Contributions

All authors contributed to the study conception and design, commented on the initial versions, read and approved the final manuscript. AV and RC were performed the material preparation, data collection, and analyses. AV wrote the first draft of the manuscript.

## Conflict of Interest

The authors declare that the research was conducted in the absence of any commercial or financial relationships that could be construed as a potential conflict of interest.

## Publisher’s Note

All claims expressed in this article are solely those of the authors and do not necessarily represent those of their affiliated organizations, or those of the publisher, the editors and the reviewers. Any product that may be evaluated in this article, or claim that may be made by its manufacturer, is not guaranteed or endorsed by the publisher.

## Abbreviations

P scale: Positivity scale
RSES: Rosenberg Self-Esteem Scale
GHQ: General Health Questionnaire
SWLS: satisfaction with life scale
BFI: big five inventory
CBT: cognitive behavior therapy.
