# Supplementary material for: Validation of the French Version of the Positivity Scale (P Scale)
Source: Front Psychol. 2022 Feb 2;12:724253. doi: 10.3389/fpsyg.2021.724253 (PMC8847605; doi:10.3389/fpsyg.2021.724253)
Supplement: Supplementary file 1 [file Data_Sheet_1.doc]

**Supplemental Material 1.** French version of the Positivity scale (P scale).

**Échelle P.** Pour chacune des affirmations suivantes, indiquez votre degré d’accord/de désaccord sur une échelle de 1 (désaccord maximum) à 5 (accord maximum), en cochant la case correspondant à votre opinion. Lisez attentivement les affirmations, et essayez de répondre avec la spontanéité maximale. Il n’existe pas de réponse juste ou erronée.

| **Je suis en fort désaccord** | **Je suis en partie en désaccord** | **Je ne suis ni d’accord, ni en désaccord** | **Je suis d’accord en partie** | | | **Je suis fortement d’accord** | | |
| --- | --- | --- | --- | --- | --- | --- | --- | --- |
| **1** | **2** | **3** | **4** | | | **5** | | |
| 1. J’ai une grande confiance dans l’avenir | | | | 1 | 2 | 3 | 4 | 5 |
| 2. Je suis satisfait(e) de ma vie | | | | 1 | 2 | 3 | 4 | 5 |
| 3. Quand j’en ai besoin, j’ai en général quelqu’un sur qui compter | | | | 1 | 2 | 3 | 4 | 5 |
| 4. Je regarde le futur avec espoir et enthousiasme | | | | 1 | 2 | 3 | 4 | 5 |
| 5. Globalement, je suis satisfait(e) de moi | | | | 1 | 2 | 3 | 4 | 5 |
| 6. Parfois le futur me semble obscur | | | | 1 | 2 | 3 | 4 | 5 |
| 7. Je pense que je peux être fier(fière) de beaucoup de choses | | | | 1 | 2 | 3 | 4 | 5 |
| 8. De façon générale, j’ai beaucoup de confiance en moi | | | | 1 | 2 | 3 | 4 | 5 |
